# Genetic variants of *SLC17A1* are associated with cholesterol homeostasis and hyperhomocysteinaemia in Japanese men

**DOI:** 10.1038/srep15888

**Published:** 2015-11-03

**Authors:** Teruhide Koyama, Daisuke Matsui, Nagato Kuriyama, Etsuko Ozaki, Keitaro Tanaka, Isao Oze, Nobuyuki Hamajima, Kenji Wakai, Rieko Okada, Kokichi Arisawa, Haruo Mikami, Keiichi Shimatani, Akie Hirata, Naoyuki Takashima, Sadao Suzuki, Chisato Nagata, Michiaki Kubo, Hideo Tanaka

**Affiliations:** 1 Department of Epidemiology for Community Health and Medicine, Kyoto Prefectural University of Medicine, Kyoto, Japan; 2 Department of Preventive Medicine, Faculty of Medicine, Saga University, Saga, Japan; 3 Division of Epidemiology and Prevention, Aichi Cancer Center Research Institute, Nagoya, Japan; 4 Department of Healthcare Administration, Nagoya University Graduate School of Medicine, Nagoya, Japan; 5 Department of Preventive Medicine, Nagoya University Graduate School of Medicine, Nagoya, Japan; 6 Department of Preventive Medicine, Institute of Health Biosciences, The University of Tokushima Graduate School, Tokushima, Japan; 7 Division of Cancer Registry, Prevention and Epidemiology, Chiba Cancer Center, Chiba, Japan; 8 Department of International Island and Community Medicine, Kagoshima University Graduate School of Medical and Dental Science, Kagoshima, Japan; 9 Department of Geriatric Medicine, Graduate School of Medical Sciences, Kyushu University, Fukuoka, Japan; 10 Department of Public Health, Shiga University of Medical Science, Otsu, Japan; 11 Department of Public Health, Nagoya City University Graduate School of Medical Sciences, Nagoya, Japan; 12 Department of Epidemiology and Preventive Medicine, Gifu University Graduate School of Medicine, Gifu, Japan; 13 Laboratory for Genotyping Development, Center for Genomic Medicine, RIKEN, Yokohama, Japan

## Abstract

Hyperuricaemia is an undisputed and highly predictive biomarker for cardiovascular risk. *SLC17A1*, expressed in the liver and kidneys, harbours potent candidate single nucleotide polymorphisms that decrease uric acid levels. Therefore, we examined *SLC17A1* polymorphisms (rs1165196, rs1179086, and rs3757131), which might suppress cardiovascular risk factors and that are involved in liver functioning, via a large-scale pooled analysis of the Japanese general population in a cross-sectional study. Using data from the Japan Multi-Institutional Collaborative Cohort Study, we identified 1842 participants of both sexes, 35–69-years-old, having the requisite data, and analysed their SLC17A1 genotypes. In men, logistic regression analyses revealed that minor alleles in SLC17A1 polymorphisms (rs1165196 and rs3757131) were associated with a low-/high-density lipoprotein cholesterol ratio >2.0 (rs1165196: odds ratio [OR], 0.703; 95% confidence interval [CI], 0.536–0.922; rs3757131: OR, 0.658; 95% CI, 0.500–0.866), and with homocysteine levels of >10.0 nmol/mL (rs1165196: OR, 0.544; 95% CI, 0.374*–*0.792; rs3757131: OR, 0.509; 95% CI, 0.347–0.746). Therefore, these polymorphisms had dominant negative effects on cholesterol homeostasis and hyperhomocysteinaemia, in men, independent of alcohol consumption, physical activity, or daily energy and nutrition intake. Thus, genetic variants of SLC17A1 are potential biomarkers for altered cholesterol homeostasis and hyperhomocysteinaemia in Japanese men.

Cardiovascular diseases, including coronary heart disease, stroke, and peripheral arterial disease, are the most prevalent conditions and leading causes of mortality, worldwide. In addition, age, sex, overweight, hypertension, smoking, and diabetes are widely accepted as major risk factors for the development of cardiovascular disease^1^. Furthermore, recent studies have reported that hyperuricaemia^2^,^3^,^4^ and elevated levels of homocysteine^5^,^6^ are also associated with cardiovascular disease, and a significant positive correlation has been observed between the serum concentrations of uric acid and homocysteine^7^.

In addition to the above-mentioned factors, altered lipid metabolism, such as the disruption of cholesterol homeostasis via altered liver function, is another major risk factor for cardiovascular disease^8^. Cholesterol is a critical lipid that is a component of biological cell membranes and is an important precursor of steroid hormones and bile acids. Unfortunately, the disruption of cholesterol homeostasis significantly increases the risk of premature cardiovascular disease^9^. To monitor for this disruption, low-density lipoprotein cholesterol (LDL-C) and high-density lipoprotein cholesterol (HDL-C) are among the most commonly used clinical biomarkers^10^, with high LDL-C and low HDL-C levels indicating major risks for the development of coronary heart disease^11^. In addition, prospective studies investigating different racial and ethnic populations have confirmed that HDL-C is a strong, consistent, and independent predictive factor for the incidence of cardiovascular disease^10^,^12^. Furthermore, the Framingham study demonstrated that a combination of high LDL-C and low HDL-C levels is a strong predictor of the relative risk of cardiovascular events^13^. Moreover, the LDL-C/HDL-C ratio is a precise marker of cholesterol homeostasis, and can help predict cardiovascular events^14^.

Previous genome-wide association studies have examined the genetic factors that control serum uric acid concentrations, and have identified 14 candidate causal single nucleotide polymorphisms (SNPs) and two pathways involving the *PKD2*, *SLC17A1*, *SLC17A3*, *SLC17A4*, and *SLC2A9* genes^15^. One of these genes (*SLC17A1*) encodes the solute carrier family 17 (organic anion transporter), member 1, which is also known as sodium phosphate transport protein 1. This protein is expressed in the sinusoidal membrane of hepatocytes and in the proximal tubules in the kidneys^16^; in the proximal tubules, it acts as a renal transporter of uric acid and mediates the co-transport of sodium and inorganic phosphate^17^. Additionally, *SLC17A1* SNPs are associated with kidney function indicators, such as suppressed serum uric levels and gout, in Japanese men^18^. Because hyperuricaemia is an undisputed and highly effective biomarker for predicting cardiovascular risk, *SLC17A1* SNPs might also suppress cardiovascular risk factors. However, whether *SLC17A1* SNPs, associated with liver function, are also associated with cardiovascular risk factors remains unknown. Cholesterol and homocysteine are both metabolised in the liver; therefore, we analysed three *SLC17A1* SNPs (rs1165196, rs1179086, and rs3757131) to evaluate their association with cholesterol homeostasis and homocysteine levels, and their roles as predictors of cardiovascular events. This analysis was conducted as a cross-sectional study using a large-scale pooled analysis of the Japanese general population.

## Results

### Participant characteristics

Table 1 shows the participants’ characteristics, including their anthropometric measures, blood chemistry data, questionnaire responses, total energy and macronutrient intake, and distribution of the three polymorphisms according to sex. The mean age of the included men was 55.6 years, compared to 54.8 years for the women. Significant differences were not observed in comparisons of the metabolic equivalents (METs) and polymorphism allele frequency data between the men and women. Furthermore, the allele frequencies for the polymorphisms were similar to those in other Japanese populations^18^, and were in agreement with the Hardy-Weinberg equilibrium (rs1165196 for men: χ^2^ = 0.571, p = 0.319; rs1165196 for women: χ^2^ = 1.241, p = 0.265; rs1179086 for men: χ^2^ = 0.023, p = 0.877; rs1179086 for women: χ^2^ = 1.350, p = 0.245; rs3757131 for men: χ^2^ = 0.284, p = 0.593; rs3757131 for women: χ^2^ = 1.053, p = 0.304).

### Associations between polymorphisms and participant characteristics, according to sex

The distributions of the three polymorphism genotypes are listed in Tables 2-4. For each genotype, the major homozygotes had significantly higher levels of uric acid, compared to the heterozygotes and minor homozygotes, in both sexes. Similarly, the major homozygous alleles in rs1165196 and rs3757131 exhibited high homocysteine and low folic acid levels in men. We also compared the major homozygotes with the heterozygotes and minor homozygotes. In men, rs1165196 and rs3757131 exhibited similar statistical significance. When compared to the major homozygous alleles, the combined minor genotype (TC + CC in rs1165196 and CT + TT in rs3757131) was associated with significantly higher folic acid levels, and significantly lower uric acid and homocysteine levels. In contrast, women did not demonstrate any significant variables associated with uric acid and homocysteine levels in each genotype.

### Associations of polymorphism genotypes with LDL-C/HDL-C ratios and homocysteine levels

Table 5 shows the proportion of participants with LDL-C/HDL-C ratios ≤2.0 and those >2.0, according to the polymorphism genotypes. For the logistic regression analysis, the major homozygous genotypes were used as the reference group and the heterozygous and minor homozygous genotypes were used as the exposed group in the dominant model. When we combined the TC and CC genotypes for rs1165196 as the low-risk genotype comparison group (assuming a dominant effect for the variant C allele), the combined genotype (TC + CC) in men was associated with a significantly lower proportion of participants with an LDL-C/HDL-C ratio of >2.0 (OR, 0.703; 95% CI, 0.536–0.922; OR adjusted for age, body mass index [BMI], research area, alcohol consumption and smoking habits, 0.642; 95% CI, 0.483–0.853), relative to the TT genotype. The combined genotype (CT + TT) in rs3757131 was also associated with a significantly lower proportion of participants with an LDL-C/HDL-C ratio >2.0 (OR, 0.658; 95% CI, 0.500–0.866; OR adjusted for age, BMI, research area, alcohol consumption and smoking habits, 0.607; 95% CI, 0.455–0.808), relative to the major homozygous alleles. When we compared the rs1165196 and rs3757131 alleles in men using an LDL-C/HDL-C ratio of >2.0, rs1165196 had a significantly lower frequency of the C allele, whereas rs3757131 had a significantly lower frequency of the T allele (T vs. C alleles in rs1165196: OR, 0.726; 95% CI, 0.573–0.920; C vs. T alleles in rs3757131: OR, 0.689; 95% CI, 0.542–0.876). Furthermore, we found that physical activity and daily energy or nutritional intake did not affect the LDL-C/HDL-C ratio in rs1165196 or rs3757131. In contrast, the proportions of women with an LDL-C/HDL-C ratio of >2.0 were not significantly different when the various polymorphism genotypes were compared.

Similar analyses were performed for homocysteine levels (Table 6). In men, when we compared the alleles for rs1165196 and rs3757131 using homocysteine levels of >10.0 nmol/mL, rs1165196 had a significantly lower frequency of the C allele, whereas rs3757131 had a significantly lower frequency of the T allele (T vs. C alleles in rs1165196: OR, 0.589; 95% CI, 0.420–0.826; C vs. T alleles in rs3757131: OR, 0.564; 95% CI, 0.399–0.797). Moreover, we confirmed that the tendency attenuated the relationship between hyperhomocysteinaemia and the *SLC17A1* polymorphisms, after adjusting for folic acid levels. In contrast, the proportion of women with homocysteine levels >10.0 nmol/mL were not significantly different when we compared the various polymorphism genotypes.

## Discussion

High levels of uric acid are historically associated with gout, although recent studies have revealed that they might also be associated with cardiovascular disease and the incidence of coronary heart disease, hypertension, stroke, metabolic syndrome, and other disorders. Therefore, patients presenting with high uric acid levels should be screened and treated for comorbid cardiovascular risk factors^19^. Furthermore, a previous genome-wide association study of uric acid transporters revealed that several SNPs were significant genetic determinants of uric acid levels^20^. In that study, the *SLC17A1* polymorphisms exhibited an association with low serum levels of uric acid. The polymorphisms of interest for *SLC17A1* are rs1165196, rs1179086, and rs3757131; these polymorphisms are known to be associated with low levels of uric acid in Japanese men^18^. However, because these three SNPs exhibit strong linkage disequilibrium^18^, determining which SNP is responsible for the functional changes in SLC17A1 is difficult. In agreement with these previous studies, our findings revealed significant associations between the *SLC17A1* polymorphisms and uric acid levels in Japanese men. Furthermore, in the present study, we also compared the associations between various *SLC17A1* genotypes and cardiovascular risk factors via altered cholesterol homeostasis and hyperhomocysteinaemia, after combining the heterozygous and minor homozygous alleles due to the small number of minor homozygotes. Our results indicated that, among male participants, the presence of minor alleles in rs1165196 and rs3757131 were associated with significantly lower homocysteine levels, compared to the major alleles. In addition, the odds of having a high LDL-C/HDL-C ratio or hyperhomocysteinaemia were significantly lower for patients with the minor alleles (vs. the major homozygous alleles), both before and after adjusting for age, BMI, research area, daily energy and nutritional intake, and alcohol consumption and smoking habits.

In healthy humans, uric acid is excreted in the urine, although kidney disease can impair this excretion route, leading to hyperuricaemia. In addition, hyperuricaemia can be caused by the increased generation of uric acid. Furthermore, exposure to lead or diets involving excessive intake of alcohol, purine nucleotides, protein, and carbohydrates can also contribute to high levels of uric acid^3^. However, in the present study, we found that alcohol consumption, physical activity, and daily energy or nutritional intake were not different for each SNP. Another potential mechanism leading to high uric acid levels is mutation(s) in the genes coding for the glucokinase regulatory protein; PDZ domain-containing proteins; and transporters of organic anions, glucose, ATP-binding cassettes, or monocarboxylic acid^15^,^20^,^21^; these transport proteins are key regulators of uric acid levels. Interestingly, *SLC17A1* is the gene involved in the renal transport of uric acid, and has been associated with low uric acid levels^18^.

HDL-C is an undisputed and highly predictive biomarker of cardiovascular risk. In this context, HDL-C is central to reverse cholesterol transport, which is the cardioprotective mechanism by which cholesterol, synthesized in the peripheral tissues, is transported to the liver for degradation or recycling. Various factors can result in elevated HDL-C levels, including smoking, alcohol consumption, exercise, and statin therapy. However, epidemiological studies have demonstrated that uric acid levels are inversely correlated with HDL-C levels^22^, and that there is a strong relationship between LDL-C levels and the incidence of atherosclerotic cardiovascular disease^10^,^23^. Another recent study has demonstrated that the LDL-C/HDL-C ratio is a precise marker of cholesterol homeostasis, and can be used to predict the risk of cardiovascular events^24^. Nicholls *et al.* showed that an LDL-C/HDL-C ratio >2.0 was associated with plaque progression (despite statin usage), whereas a ratio <1.5 was significantly associated with plaque regression^25^. Many studies have indicated that cholesterol homeostasis is a major mechanism for suppressing cardiovascular disease. In the present study, we used the LDL-C/HDL-C ratio to evaluate cholesterol homeostasis because it is a better predictor of future cardiovascular disease than are single lipid parameters (e.g., triglyceride, LDL-C, or HDL-C levels)^14^. However, we did not find a correlation between uric acid levels and cholesterol homeostasis for minor alleles in rs1165196 and rs3757131 (data not shown). These results suggest that the minor alleles in rs1165196 and rs3757131 had independent effects on uric acid levels and cholesterol homeostasis.

Various cardiovascular disease risk factors are reported to be related to homocysteine levels, including total cholesterol, other lipids, smoking, and the presence or absence of hypertension and diabetes mellitus. However, adjusting for these risk factors only weakly attenuates the strong relationship between homocysteine levels and mortality due to cardiovascular causes^5^. In addition, homocysteine levels are higher in patients with gout than in healthy controls, and uric acid levels are known to be correlated with homocysteine levels^22^. In contrast, hyperhomocysteinaemia is not correlated with uric acid levels in patients with gout, although there is an inverse association between homocysteine levels and renal function^26^. These reports indicate that hyperhomocysteinaemia, which may be related to uric acid levels and cardiovascular mortality, is clearly a cardiovascular risk factor, although the precise relationship remains unknown.

Interestingly, an association between hyperlipidaemia and hyperhomocysteinaemia has been suggested. In an animal model, hyperhomocysteinaemia inhibited reverse cholesterol transport by reducing circulating HDL levels, via inhibition of apoA-I protein synthesis, and enhanced HDL-C clearance^27^. Therefore, the liver may be a major organ involved in regulating homocysteine and cholesterol homeostasis^28^. These studies suggest that the interactions between homocysteine and HDL metabolism may be clinically important. Based on our findings, the rs1165196 and rs3757131 polymorphisms may be suitable biomarkers for cardiovascular disease risk factors, although their direct effect(s) on the metabolic pathway remains unknown.

Plasma homocysteine concentrations are typically considered to be inversely related to folic acid levels (a cofactor or substrate for enzymes involved in homocysteine metabolism). Similarly, a previous study reported that homocysteine levels in elderly Japanese individuals are inversely related to folic acid levels^29^. Furthermore, another study reported that folic acid supplementation lowered homocysteine levels, although the change in lipid metabolism was not significant at the end of the treatment period^28^,^30^. Nevertheless, the preventative effects of folic acid supplementation on cardiovascular disease cannot be excluded^31^. In the present study, high homocysteine and low folic acid levels were associated with the major homozygous alleles in rs1165196 and rs3757131. However, adjusting for folic acid weakly attenuated the relationship between hyperhomocysteinaemia and the *SLC17A1* polymorphisms. Nevertheless, the odds of hyperhomocysteinaemia were significantly different for rs1165196 and rs3757131, even after adjusting for folic acid levels. Therefore, *SLC17A1* polymorphisms appear to independently affect low homocysteine levels.

To the best of our knowledge, the present study was the first to explore the association between rs1165196 and rs3757131 genotypes and cardiovascular risk factors, altered cholesterol homeostasis, and homocysteine levels in Japanese men. However, in women, statistically significant differences in cholesterol homeostasis and homocysteine levels were not identified. Similarly, pronounced sex-related differences in the regulation of uric acid levels have been reported in both humans and animals^32^. In the Framingham Heart Study, the association between uric acid levels and mortality risk in men was not significant in the univariate analysis, and the association in women was not significant after adjusting for diuretic use, blood pressure, and total cholesterol levels^33^. Therefore, the specific physiological characteristics of women may be related to the sex-related differences in SNP genotypes that were observed to be associated with uric acid levels, although the underlying mechanism has yet to be elucidated. Furthermore, alcohol consumption, physical activity, and daily energy and nutrition intake did not affect the associations in either men or women. Therefore, identification of the pathogenic genetic factor(s) for cardiovascular disease remains critically important.

The limitations of our study include its cross-sectional design and the relatively small number of participants. Although a study with a low statistical power has a reduced likelihood of detecting a true effect, case-control studies with small sample sizes are still widely used, and can be used to assess previously identified candidate regions and more precisely determine target selections. In addition, we only assessed Japanese participants in the present study, and further studies in other ethnic groups are needed to validate our findings. Therefore, large, prospective trials involving patients from multiple ethnic groups are needed to better assess the effects of *SLC17A1* polymorphisms on cholesterol homeostasis and hyperhomocysteinaemia.

## Conclusion

In conclusion, our results indicate that the rs1165196 and rs3757131 polymorphisms, expressed in the liver, confer dominant negative effects on LDL-C/HDL-C ratios and hyperhomocysteinaemia in Japanese men. Although the exact biological mechanism for this association remains unknown, our findings provide credible evidence that the rs1165196 and rs3757131 polymorphisms may be associated with a decreased risk of cardiovascular disease. This risk reduction may be due to their important role in maintaining cholesterol homeostasis and preventing hyperhomocysteinaemia through altered liver function. However, further studies are needed to interpret the effects of *SLC17A1* polymorphisms on cardiovascular disease.

## Methods

### Study participants

In the present study, we evaluated participant data collected during the Japan Multi-Institutional Collaborative Cohort (J-MICC) Study. That cohort study evaluated the general Japanese population in 10 research areas, using genetic and clinical data to detect and confirm gene-environment interactions related to lifestyle-associated diseases^34^. The study participants were 35–69 years old, and were enrolled after responding to study announcements in their specific research areas, attending health check-up examinations that were commissioned by their local governments, visiting local health check-up centres, or visiting a cancer hospital. A total of 4490 participants were selected, with approximately 500–600 participants from each research area, except for two areas in which fewer participants were recruited^35^.

Among these 4490 participants, 2035 were excluded because they had consumed a meal <6 h before having their blood drawn, 228 because they were receiving cholesterol-lowering medication, 24 due to high triglyceride levels (≥400 mg/dL), 338 due to insufficient laboratory data, and 23 individuals were excluded due to the absence of other data. After these exclusions, 1842 individuals (995 men and 847 women) were eligible for analyses. Folic acid (751 men and 620 women) and homocysteine (719 men and 606 women) levels limited the number of participants.

All participants provided written informed consent for the J-MICC Study, and the main study protocol of the J-MICC Study was approved by Ethics Committee at Nagoya University School of Medicine (Approval number 253 and 939). Since actual studies are slightly different from the main J-MICC Study protocol, all procedures involved in this study were performed in accordance with the institutional ethical committees (Chiba Cancer Center, Nagoya University, Nagoya City University, Aichi Cancer Center, Shiga University of Medical Science, Kyoto Prefectural University of Medicine, Tokushima University, Kyushu University, Saga University, and Kagoshima University).

### Blood biochemistry, lifestyle, and nutritional data

In the present study, we evaluated lifestyle and medical information obtained through self-administered questionnaires (alcohol consumption status [0, 0.1–22.9, 23.0–45.9, or ≥46.0 g ethanol/day], smoking habits, current medications, and exercise). In addition, blood chemistry data (serum levels of triglycerides, total cholesterol, HDL-C, uric acid, LDL-C [calculated using the Friedewald formula^36^, and plasma levels of homocysteine and folic acid) and anthropometric data were obtained from health check-ups performed in the research areas. Each blood sample was centrifuged and the plasma was separated and stored at –80 °C until analysis. Serum samples were measured by laboratories in each research area. Plasma folic acid concentrations were measured using a chemiluminescent enzyme immunoassay, and plasma homocysteine concentrations were measured using high-performance liquid chromatography by a contract laboratory (SRL, Tokyo, Japan).

For the dietary assessment, a validated food-frequency questionnaire was used to evaluate the intake frequency of 47 foods and beverages over the preceding year, and the total daily energy (kcal), protein (g), fat (g), and carbohydrate (g) intakes were calculated^37^,^38^,^39^,^40^. Physical activity was assessed as METs for daily and leisure activities, as previously reported^41^. In brief, METs-hours per day (METs·h/day) of daily activity were estimated for heavy physical work and walking. For leisure activities, METs·h/day were estimated by multiplying the reported daily time spent in each activity by the relevant MET intensity.

### Genotyping *SLC17A1* polymorphisms

Our previous study described the details of the genetic analysis^35^. Briefly, genomic DNA was extracted from the buffy coat fraction of the participant’s blood sample, using a BioRobot M48 Workstation (QIAGEN Group, Tokyo, Japan). The *SLC17A1* polymorphisms (rs1165196, rs1179086, and rs3757131) were genotyped using a multiplex polymerase chain reaction-based Invader assay (Third Wave Technologies, Madison, WI, USA) at the RIKEN Laboratory for Genotyping Development, Center for Genomic Medicine (Yokohama, Japan)^42^. The rs1165196 polymorphism is located at exon 7, where a conversion from thymine to cytosine at nucleotide 806 results in the substitution of threonine for isoleucine. The rs1179086 and rs3757131 polymorphisms are located at intron 12^18^.

### Statistical analyses

Inter-group comparisons were performed using Student’s unpaired *t-*tests for continuous variables and chi-square tests for categorical variables (alcohol consumption in current drinkers, smokers, and allele independence [Hardy-Weinberg equilibrium]). Differences in quantitative data, between the genotypes, were evaluated using one-way analysis of variance. All data were expressed as means ± standard deviation, or as indicated, and the coefficients of variation (CVs) for each parameter were calculated. ORs, 95% CIs, and p-values were calculated using logistic regression analyses; the LDL-C/HDL-C ratio (>2.0) was defined as the dependent variable, and participant age, sex, BMI (kg/m^2^), research area, METs, daily energy and nutritional intake (protein, fat, and carbohydrate), and alcohol consumption and smoking habits, were included as independent variables. In the general Japanese population, a plasma homocysteine concentration of >10.0 nmol/mL is defined as hyperhomocysteinaemia^29^, and this cut-off value was used for our analyses. We performed logistic regression analyses with the homocysteine level defined as the dependent variable, and patient age, sex, BMI, research area, folic acid level, and alcohol consumption and smoking habits were included as independent variables. All statistical tests were two-sided, and differences with a p-value of <0.05 were considered statistically significant. SPSS software (version 18.0, SPSS, Japan, Inc.) was used for all statistical analyses.

## Additional Information

**How to cite this article**: Koyama, T. *et al.* Genetic variants of *SLC17A1* are associated with cholesterol homeostasis and hyperhomocysteinaemia in Japanese men. *Sci. Rep.* **5**, 15888; doi: 10.1038/srep15888 (2015).

## Figures and Tables

**Table 1:** Clinical characteristics and genotype frequencies of the participants, according to sex.

	Men (n = 995)		Women (n = 847)		
	Mean ± SD (%)	CV (%)	Mean ± SD (%)	CV (%)	p-value
Age (years)	55.6 ± 8.8	15.8	54.8 ± 8.5	15.5	0.052
BMI (kg/m^2^)	23.8 ± 3.1	13.0	22.9 ± 3.3	14.4	<0.001
Triglycerides (mg/dL)	120.7 ± 61.0	50.5	92.2 ± 49.3	53.4	<0.001
Total cholesterol (mg/dL)	204.3 ± 31.4	15.3	216.9 ± 35.4	16.3	<0.001
LDL-C (mg/dL)	120.6 ± 29.7	24.6	129.5 ± 33.7	26.0	<0.001
HDL-C (mg/dL)	59.5 ± 15.7	26.3	68.9 ± 15.2	22.0	<0.001
LDL-C/HDL-C	2.17 ± 0.78	35.9	2.00 ± 0.77	38.5	<0.001
Uric acid (mg/dL)	6.03 ± 1.17	19.4	4.43 ± 0.99	22.3	<0.001
Folic acid (ng/mL)*	8.61 ± 2.25	26.1	9.75 ± 3.26	33.4	<0.001
Homocysteine (nmol/mL)**	9.37 ± 3.48	37.1	7.36 ± 2.51	34.1	<0.001
Energy intake (kcal/day)	1,930 ± 360	18.6	1,547 ± 251	16.2	<0.001
Protein intake (g/day)	55.8 ± 11.2	20.0	51.3 ± 10.5	20.4	<0.001
Fat intake (g/day)	41.8 ± 10.5	25.1	44.6 ± 11.7	26.2	<0.001
Carbohydrate intake (g/day)	278.6 ± 70.3	25.2	216.4 ± 45.1	20.8	<0.001
METs (h/day)	14.7 ± 14.0	95.2	15.0 ± 13.7	91.3	0.693
Alcohol drinking					
0 g/d	393 (39.4)		593 (70.0)		
0.1–22.9 g/d	414 (41.6)		238 (28.0)		<0.001
23.0–45.9 g/d	122 (12.2)		15 (1.77)		
≥46.0 g/d	66 (6.63)		1 (0.11)		
Smoking					
Current	272 (27.3)		45 (5.31)		<0.001
Former	433 (43.5)		25 (2.95)		
Never	290 (29.1)		777 (91.7)		
rs1165196					
TT	694 (69.7)		612 (72.2)		0.496
TC	271 (27.2)		211 (24.9)		
CC	30 (3.01)		24 (2.83)		
rs1179086					
AA	542 (54.4)		467 (55.1)		0.634
AT	386 (38.7)		315 (37.1)		
TT	67 (6.73)		65 (7.67)		
rs3757131					
CC	703 (70.6)		610 (72.0)		0.795
CT	264 (26.5)		213 (25.1)		
TT	28 (2.81)		24 (2.83)		

Data are means ± standard deviation.

CV, coefficient of variance BMI, body mass index; LDL-C, low-density lipoprotein cholesterol.

HDL-C, high-density lipoprotein cholesterol; METs, metabolic equivalents.

Sex-related differences were analysed using the *t*-test,

Chi-square tests were used for smoking and alcohol habits and genotypes.

^*^Men = 751, Women = 620, **Men = 719, Women = 606.

**Table 2:** The distribution of the participants, according to sex, for rs1165196.

Sex	Men						Women					
Genotype (N)	TT (694)	TC (271)	CC (30)	p*	TC + CC	p** (vs. TT)	TT (612)	TC (211)	CC (24)	p*	TC + CC	p** (vs. TT)
Age (years)	55.5 ± 8.8	55.5 ± 8.9	58.2 ± 7.9	0.271	55.7 ± 8.8	0.730	54.7 ± 8.7	54.8 ± 8.1	58.2 ± 7.0	0.131	55.1 ± 8.1	0.491
BMI (kg/m^2^)	23.7 ± 3.0	24.1 ± 3.1	23.7 ± 2.9	0.145	24.1 ± 3.1	0.062	22.9 ± 3.3	22.7 ± 3.2	23.5 ± 4.1	0.483	22.8 ± 3.3	0.570
Triglycerides (mg/dL)	120.6 ± 59.4	121.8 ± 64.9	112.8 ± 64.2	0.741	120.9 ± 64.8	0.946	93.4 ± 50.0	88.3 ± 45.8	94.0 ± 59.5	0.433	88.9 ± 47.3	0.238
Total cholesterol (mg/dL)	204.2 ± 31.4	204.0 ± 31.5	211.0 ± 32.1	0.503	204.7 ± 31.6	0.818	217.7 ± 35.1	214.0 ± 36.9	222.0 ± 27.1	0.324	214.8 ± 36.0	0.287
LDL-C (mg/dL)	121.1 ± 29.9	119.3 ± 29.2	121.5 ± 30.0	0.690	119.5 ± 29.2	0.441	129.8 ± 33.1	128.1 ± 36.0	133.8 ± 27.1	0.668	128.7 ± 35.2	0.665
HDL-C (mg/dL)	58.9 ± 15.5	60.3 ± 16.0	66.8 ± 14.3	0.017	60.9 ± 16.0	0.062	69.1 ± 15.4	68.1 ± 15.0	69.4 ± 11.4	0.705	68.3 ± 14.6	0.454
LDL-C/HDL-C	2.20 ± 0.79	2.12 ± 0.75	1.92 ± 0.71	0.069	2.10 ± 0.75	0.053	2.00 ± 0.77	2.00 ± 0.81	1.99 ± 0.58	0.999	2.00 ± 0.79	0.977
Uric acid (mg/dL)	6.10 ± 1.14	5.87 ± 1.21	5.94 ± 1.40	0.020	5.88 ± 1.23	0.005	4.49 ± 1.00	4.27 ± 0.92	4.28 ± 1.22	0.016	4.27 ± 0.95	0.004
Folic acid (ng/mL)*	8.42 ± 2.13	8.98 ± 2.51	9.69 ± 1.80	0.001	9.04 ± 2.46	0.001	9.71 ± 3.08	9.84 ± 3.74	9.84 ± 2.97	0.912	9.84 ± 3.66	0.667
Homocysteine (nmol/mL)**	9.66 ± 3.85	8.70 ± 2.34	8.51 ± 1.53	0.003	8.69 ± 2.29	<0.001	7.36 ± 2.72	7.38 ± 1.92	7.20 ± 1.92	0.957	7.36 ± 1.91	0.982
Energy intake (kcal/day)	1,929 ± 356	1,933 ± 376	1,919 ± 284	0.976	1,931 ± 367	0.925	1,546 ± 248	1,552 ± 257	1,542 ± 267	0.957	1,551 ± 258	0.813
Protein intake (g/day)	56.0 ± 11.4	55.2 ± 11.0	55.1 ± 9.52	0.587	55.2 ± 10.9	0.303	51.4 ± 10.6	51.4 ± 10.3	50.2 ± 12.3	0.866	51.3 ± 10.5	0.893
Fat intake (g/day)	41.9 ± 10.8	41.4 ± 9.89	43.1 ± 8.78	0.654	41.6 ± 9.78	0.676	44.5 ± 11.7	45.0 ± 11.7	45.1 ± 11.8	0.830	45.0 ± 11.7	0.542
Carbohydrate intake (g/day)	279.0 ± 71.1	278.8 ± 69.8	267.8 ± 56.8	0.695	277.7 ± 68.6	0.797	215.9 ± 43.9	218.6 ± 47.4	211.1 ± 56.6	0.630	217.9 ± 48.3	0.565
METs (h/day)	14.9 ± 14.1	14.4 ± 14.5	13.7 ± 8.7	0.847	14.4 ± 14.0	0.606	15.0 ± 13.5	15.2 ± 14.3	13.2 ± 11.4	0.790	15.0 ± 14.1	0.976

Data are presented as means ± standard deviation; BMI, body mass index; LDL-C, low-density lipoprotein cholesterol; HDL-C, high-density lipoprotein cholesterol; METs, metabolic equivalents.

Genotype differences were analysed using one-way analysis of variance^*^, or the *t*-test^**^

^*^Men (TT = 524, TC = 208, CC = 19) Women (TT= 439, TC = 161, CC = 20).

^**^Men (TT = 502, TC = 201, CC = 16) Women (TT = 431, TC = 156, CC = 19).

**Table 3:** The distribution of participants, according to sex, for rs1179086.

Sex	Men						Women					
Genotype (N)	AA (542)	AT (386)	TT (67)	p*	AT + TT	p** (vs. AA)	AA (467)	AT (315)	TT (65)	p*	AT + TT	p** (vs. AA)
Age (years)	55.3 ± 8.9	55.7 ± 8.8	56.5 ± 8.3	0.537	55.9 ± 8.7	0.371	55.1 ± 8.5	54.5 ± 8.4	54.0 ± 8.9	0.501	54.4 ± 8.5	0.289
BMI (kg/m^2^)	23.6 ± 2.9	24.0 ± 3.2	23.7 ± 2.9	0.161	24.0 ± 3.2	0.086	22.9 ± 3.3	22.8 ± 3.3	23.1 ± 3.4	0.844	22.9 ± 3.3	0.850
Triglycerides (mg/dL)	118.3 ± 58.4	125.2 ± 64.7	114.6 ± 59.6	0.168	123.6 ± 64.0	0.175	94.7 ± 52.4	87.5 ± 41.7	96.6 ± 58.6	0.099	89.0 ± 45.1	0.096
Total cholesterol (mg/dL)	204.0 ± 30.9	203.9 ± 31.8	209.1 ± 33.4	0.438	204.7 ± 32.1	0.741	218.7 ± 35.5	214.8 ± 35.7	214.2 ± 32.9	0.258	214.7 ± 35.2	0.101
LDL-C (mg/dL)	121.0 ± 29.2	119.8 ± 30.2	122.1 ± 31.2	0.769	120.2 ± 30.3	0.662	130.2 ± 33.4	129.2 ± 34.5	125.7 ± 32.2	0.599	128.6 ± 34.1	0.501
HDL-C (mg/dL)	59.3 ± 15.7	59.0 ± 15.8	64.0 ± 14.3	0.049	59.7 ± 15.7	0.662	69.5 ± 15.8	68.0 ± 14.5	69.0 ± 14.3	0.403	68.2 ± 14.4	0.212
LDL-C/HDL-C	2.19 ± 0.79	2.17 ± 0.78	2.00 ± 0.70	0.198	2.15 ± 0.77	0.417	1.99 ± 0.78	2.01 ± 0.78	1.93 ± 0.72	0.721	2.00 ± 0.77	0.964
Uric acid (mg/dL)	6.13 ± 1.18	5.94 ± 1.14	5.83 ± 1.26	0.016	5.92 ± 1.16	0.005	4.53 ± 0.98	4.31 ± 0.97	4.23 ± 1.04	0.002	4.30 ± 0.98	0.001
Folic acid (ng/mL)	8.44 ± 2.16	8.79 ± 2.39	9.05 ± 1.95	0.054	8.82 ± 2.34	0.021	9.78 ± 3.24	9.76 ± 3.37	9.53 ± 2.78	0.888	9.72 ± 3.28	0.838
Homocysteine (nmol/mL)**	9.53 ± 3.92	9.23 ± 2.95	8.63 ± 1.79	0.217	9.16 ± 2.83	0.156	7.19 ± 2.16	7.50 ± 2.98	7.86 ± 2.29	0.142	7.56 ± 2.88	0.076
Energy intake (kcal/day)	1,938 ± 350	1,921 ± 381	1,908 ± 308	0.687	1,919 ± 371	0.413	1,553 ± 253	1,543 ± 251	1,530 ± 241	0.740	1,541 ± 249	0.494
Protein intake (g/day)	56.1 ± 11.2	55.5 ± 11.4	54.4 ± 10.1	0.407	55.3 ± 11.2	0.272	51.4 ± 10.2	51.5 ± 10.9	50.4 ± 11.3	0.748	51.3 ± 11.0	0.884
Fat intake (g/day)	41.8 ± 10.8	41.6 ± 9.94	42.3 ± 11.1	0.881	41.7 ± 10.1	0.891	44.3 ± 11.1	45.0 ± 12.1	45.9 ± 13.9	0.490	45.1 ± 12.4	0.290
Carbohydrate intake (g/day)	282.4 ± 69.7	274.8 ± 72.9	269.6 ± 57.6	0.152	274.1 ± 70.9	0.063	216.8 ± 44.8	216.7 ± 45.1	212.2 ± 48.6	0.728	216.0 ± 45.7	0.777
METs (h/day)	14.8 ± 13.7	14.5 ± 14.9	15.3 ± 12.3	0.899	14.6 ± 14.5	0.844	14.6 ± 13.2	15.3 ± 14.4	16.0 ± 13.9	0.596	15.5 ± 14.3	0.342

Data are presented as means ± standard deviation; BMI, body mass index; LDL-C, low-density lipoprotein cholesterol; HDL-C, high-density lipoprotein cholesterol; METs, metabolic equivalents.

Genotype differences were analysed using one-way analysis of variance^*^, or the *t*-test^**^.

^*^Men (TT = 412, TC = 294, CC = 45) Women (TT = 335, TC = 237, CC = 48).

^**^Men (TT = 399, TC = 281, CC = 39) Women (TT = 330, TC = 231, CC = 65).

**Table 4:** The distribution of the participants, according to sex, for rs3757131.

Sex	Men						Women					
Genotype (N)	CC (703)	CT (264)	TT (28)	p*	CT+ TT	p** (vs. CC)	CC (610)	CT (213)	TT (24)	p*	CT+ TT	p** (vs. CC)
Age (years)	55.5 ± 8.8	55.4 ± 8.9	58.0 ± 7.9	0.342	55.7 ± 8.8	0.808	54.7 ± 8.7	54.9 ± 8.0	57.5 ± 7.7	0.293	55.1 ± 8.0	0.496
BMI (kg/m^2^)	23.7 ± 3.0	24.1 ± 3.2	23.6 ± 2.6	0.155	24.0 ± 3.1	0.073	22.9 ± 3.3	22.7 ± 3.2	23.3 ± 4.0	0.630	22.8 ± 3.3	0.570
Triglycerides (mg/dL)	120.6 ± 59.2	122.0 ± 65.6	111.9 ± 64.0	0.708	121.0 ± 65.4	0.932	93.1 ± 49.6	89.3 ± 47.3	94.1 ± 59.4	0.628	89.8 ± 48.5	0.394
Total cholesterol (mg/dL)	203.9 ± 31.4	204.6 ± 31.4	212.2 ± 32.8	0.391	205.3 ± 31.6	0.536	217.8 ± 35.1	213.8 ± 37.0	220.7 ± 27.7	0.310	214.5 ± 36.2	0.219
LDL-C (mg/dL)	121.0 ± 29.8	119.3 ± 29.3	122.7 ± 30.6	0.673	119.6 ± 29.4	0.501	129.9 ± 33.2	128.0 ± 35.9	131.1 ± 28.6	0.749	128.3 ± 35.2	0.530
HDL-C (mg/dL)	58.7 ± 15.4	60.8 ± 16.3	67.0 ± 14.2	0.007	61.4 ± 16.2	0.014	69.2 ± 15.4	67.8 ± 14.8	70.7 ± 12.7	0.449	68.1 ± 14.6	0.362
LDL-C/HDL-C	2.20 ± 0.79	2.10 ± 0.76	1.94 ± 0.73	0.058	2.09 ± 0.76	0.032	2.00 ± 0.77	2.00 ± 0.80	1.93 ± 0.62	0.919	1.99 ± 0.79	0.962
Uric acid (mg/dL)	6.10 ± 1.15	5.86 ± 1.21	5.95 ± 1.42	0.015	5.87 ± 1.23	0.004	4.48 ± 1.00	4.29 ± 0.92	4.19 ± 1.19	0.029	4.28 ± 0.95	0.009
Folic acid (ng/mL)	8.43 ± 2.12	9.00 ± 2.52	9.72 ± 1.85	0.001	9.05 ± 2.48	<0.001	9.70 ± 3.08	9.88 ± 3.74	9.71 ± 2.86	0.830	9.86 ± 3.65	0.572
Homocysteine (nmol/mL)**	9.67 ± 3.84	8.63 ± 2.29	8.60 ± 1.60	0.001	8.63 ± 2.25	<0.001	7.38 ± 2.73	7.34 ± 1.92	7.18 ± 1.93	0.940	7.32 ± 1.91	0.803
Energy intake (kcal/day)	1,928 ± 355	1,934 ± 379	1,923 ± 294	0.973	1,933 ± 372	0.854	1,546 ± 247	1,550 ± 260	1,553 ± 267	0.979	1,550 ± 260	0.843
Protein intake (g/day)	56.0 ± 11.4	55.2 ± 11.0	55.0 ± 9.86	0.586	55.2 ± 10.9	0.303	51.3 ± 10.5	51.4 ± 10.4	50.5 ± 12.3	0.918	51.3 ± 10.6	0.957
Fat intake (g/day)	41.8 ± 10.8	41.6 ± 9.81	42.6 ± 8.92	0.891	41.7 ± 9.71	0.918	44.5 ± 11.7	45.0 ± 11.7	46.0 ± 11.6	0.720	45.1 ± 11.7	0.483
Carbohydrate intake (g/day)	279.2 ± 70.6	278.2 ± 70.8	268.1 ± 58.8	0.711	277.2 ± 69.7	0.683	216.1 ± 43.7	217.9 ± 47.9	212.3 ± 56.8	0.794	217.3 ± 48.8	0.718
METs (h/day)	14.9 ± 14.2	14.2 ± 14.1	13.1 ± 8.82	0.649	14.1 ± 13.6	0.396	14.9 ± 13.5	15.4 ± 14.4	12.8 ± 11.5	0.669	15.1 ± 14.1	0.840

Data are presented as means ± standard deviation; BMI, body mass index; LDL-C, low-density lipoprotein cholesterol; HDL-C, high-density lipoprotein cholesterol; METs, metabolic equivalents.

Genotype differences were analysed using one-way analysis of variance^*^, or the *t*-test^**^.

^*^Men (TT = 530, TC = 204, CC = 17) Women (TT = 435, TC = 165, CC = 20).

^**^Men (TT = 507, TC = 198, CC = 14) Women (TT = 427, TC = 160, CC = 19).

**Table 5:** Associations between the SLC17A1 gene variants and the LDL-C/HDL-C ratio.

SNP/genotype	Men								Women							
	LDL-C/HDL-C ratio (N)		OR	95% CI	OR†	95% CI†	OR‡	95% CI‡	LDL-C/HDL-C ratio (N)		OR	95% CI	OR†	95% CI†	OR‡	95% CI‡
	≤2.0	>2.0							≤2.0	>2.0						
rs1165196																
TT	292	402	Reference		Reference		Reference		336	276	Reference		Reference		Reference	
TC	136	135	0.721	0.544–0.956	0.653	0.486–0.878	0.649	0.481–0.877	124	87	0.854	0.622–1.173	0.844	0.594–1.198	0.846	0.595–1.204
CC	17	13	0.555	0.266–1.162	0.549	0.253–1.191	0.572	0.261–1.253	12	12	1.217	0.538–2.753	0.920	0.376–2.251	0.978	0.399–2.401
TC+CC	153	148	0.703	0.536–0.922	0.642	0.483–0.853	0.642	0.481–0.857	136	99	0.886	0.654–1.201	0.845	0.604–1.183	0.853	0.609–1.195
T	720	939	Reference		Reference		Reference		796	639	Reference		Reference		Reference	
C	170	161	0.726	0.573–0.920	0.679	0.531–0.869	0.684	0.533–0.878	148	111	0.934	0.715–1.220	0.869	0.647–1.167	0.877	0.653–1.179
rs1179086																
AA	234	308	Reference		Reference		Reference		256	211	Reference		Reference		Reference	
AT	175	211	0.916	0.704–1.191	0.866	0.657–1.143	0.884	0.666–1.172	177	138	0.946	0.709–1.261	0.898	0.652–1.237	0.894	0.647–1.234
TT	36	31	0.654	0.393–1.089	0.671	0.391–1.152	0.689	0.399–1.190	39	26	0.809	0.477–1.372	0.677	0.375–1.222	0.668	0.369–1.209
AT+TT	211	242	0.871	0.678–1.120	0.828	0.636–1.078	0.686	0.646–1.106	216	164	0.921	0.701–1.210	0.861	0.635–1.167	0.860	0.633–1.169
A	643	827	Reference		Reference		Reference		689	560	Reference		Reference		Reference	
T	247	273	0.859	0.703–1.050	0.835	0.677–1.029	0.849	0.686–1.050	255	190	0.917	0.737–1.141	0.855	0.671–1.090	0.857	0.672–1.093
rs3757131																
CC	293	410	Reference		Reference		Reference		334	276	Reference		Reference		Reference	
CT	136	128	0.673	0.506–0.894	0.614	0.456–0.828	0.614	0.453–0.832	125	88	0.852	0.621–1.168	0.842	0.594–1.194	0.845	0.595–1.201
TT	16	12	0.536	0.250–1.150	0.537	0.242–1.189	0.563	0.251–1.265	13	11	1.024	0.452–2.322	0.782	0.320–1.909	0.823	0.335–2.021
CT+TT	152	140	0.658	0.500–0.866	0.607	0.455–0.808	0.609	0.455–0.816	138	99	0.868	0.641–1.176	0.831	0.594–1.162	0.839	0.599–1.175
C	722	948	Reference		Reference		Reference		793	640	Reference		Reference		Reference	
T	168	152	0.689	0.542–0.876	0.649	0.506–0.833	0.656	0.509–0.846	151	110	0.903	0.691–1.179	0.844	0.629–1.133	0.853	0.635–1.146

SNP, single nucleotide polymorphism; LDL-C, low-density lipoprotein cholesterol; HDL-C, high-density lipoprotein cholesterol; OR, odds ratio; CI, confidence interval.

^†^Adjusted for age, body mass index, research area, alcohol consumption, smoking habits.

^‡^Adjusted for age, body mass index, research area, METs, daily energy and nutritional intake (protein, fat, carbohydrate), and alcohol and smoking habits.

**Table 6:** Associations between the SLC17A1 gene variants and hyperhomocysteinaemia.

SNP/genotype	Men								Women							
	Homocysteine (N)		OR	95% CI	OR†	95% CI†	OR‡	95% CI‡	Homocysteine (N)		OR	95% CI	OR†	95% CI†	OR‡	95% CI‡
	≤10.0	>10.0>							≤10.0	>10.0						
rs1165196																
TT	336	166	Reference		Reference		Reference		387	44	Reference		Reference		Reference	
TC	158	43	0.551	0.375–0.810	0.505	0.340–0.751	0.560	0.371–0.844	141	15	0.936	0.505–1.734	0.923	0.492–1.731	0.942	0.498–1.781
CC	13	3	0.467	0.131–1.662	0.447	0.122–1.634	0.542	0.143–2.047	17	2	1.035	0.231–4.628	0.792	0.169–3.704	0.796	0.166–3.814
TC+CC	171	46	0.544	0.374–0.792	0.499	0.339–0.734	0.560	0.375–0.835	158	17	0.946	0.525–1.706	0.910	0.498–1.663	0.927	0.504–1.707
T	830	375	Reference		Reference		Reference		915	103	Reference		Reference		Reference	
C	184	49	0.589	0.420–0.826	0.550	0.389–0.777	0.609	0.426–0.870	175	19	0.964	0.576–1.615	0.910	0.537–1.540	0.923	0.542–1.572
rs1179086																
AA	273	126	Reference		Reference		Reference		300	30	Reference		Reference		Reference	
AT	202	79	0.847	0.606–1.185	0.788	0.557–1.113	0.854	0.595–1.227	208	23	1.106	0.625–1.958	1.028	0.571–1.851	1.041	0.574–1.888
TT	32	7	0.474	0.204–1.103	0.431	0.181–1.022	0.468	0.192–1.137	37	8	2.162	0.923–5.066	1.868	0.763–4.574	1.883	0.751–4.723
AT+TT	234	86	0.796	0.575–1.102	0.733	0.524–1.027	0.796	0.560–1.131	245	31	1.265	0.745–2.149	1.164	0.675–2.007	1.175	0.677–2.041
A	748	331	Reference		Reference		Reference		808	83	Reference		Reference		Reference	
T	266	93	0.790	0.604–1.034	0.743	0.564–0.980	0.788	0.591–1.050	282	39	1.346	0.899–2.016	1.258	0.831–1.904	1.264	0.830–1.925
rs3757131																
CC	338	169	Reference		Reference		Reference		383	44	Reference		Reference		Reference	
CT	158	40	0.506	0.342–0.750	0.466	0.311–0.698	0.514	0.338–0.781	145	15	0.900	0.486–1.668	0.902	0.481–1.691	0.926	0.490–1.750
TT	11	3	0.545	0.150–1.981	0.536	0.144–1.997	0.665	0.171–2.584	17	2	1.024	0.229–4.580	0.763	0.163–3.569	0.763	0.160–3.467
CT+TT	169	43	0.509	0.347–0.746	0.468	0.316–0.694	0.523	0.348–0.786	162	17	0.913	0.507–1.646	0.890	0.487–1.625	0.910	0.495–1.674
C	834	378	Reference		Reference		Reference		911	103	Reference		Reference		Reference	
T	180	46	0.564	0.399–0.797	0.529	0.371–0.754	0.585	0.406–0.843	179	19	0.939	0.561–1.571	0.893	0.528–1.510	0.907	0.532–1.544

SNP, single nucleotide polymorphism; OR, odds ratio; CI, confidence interval.

^†^Adjusted for age, body mass index, research area, alcohol consumption, smoking habits.

^‡^Adjusted for age, body mass index, research area, folic acid, alcohol consumption, smoking habits.

## References

[b1] World Health Organization. *Prevention of cardiovascular disease: guidelines for assessment and management of total cardiovascular risk*. (2007). Available at: http://www.who.int/cardiovascular_diseases/guidelines/Full text.pdf. (Accessed: 21st April 2015).

[b2] Jin M. *et al.* Uric acid, hyperuricemia and vascular diseases. Front. Biosci. (Landmark Ed). 17, 656–669 (2012). 10.2741/3950 PMC3247913 22201767

[b3] Grassi D. *et al.* Chronic hyperuricemia, uric acid deposit and cardiovascular risk. Curr. Pharm. Des. 19, 2432–2438 (2013). 23173592 10.2174/1381612811319130011 PMC3606968

[b4] Kim S. Y. *et al.* Hyperuricemia and risk of stroke: a systematic review and meta-analysis. Arthritis Rheum. 61, 885–892 (2009). 19565556 10.1002/art.24612 PMC2714267

[b5] Nygard O. *et al.* Plasma homocysteine levels and mortality in patients with coronary artery disease. N. Engl. J. Med. 337, 230–236 (1997). 9227928 10.1056/NEJM199707243370403

[b6] Okura T. *et al.* Hyperhomocysteinemia is one of the risk factors associated with cerebrovascular stiffness in hypertensive patients, especially elderly males. Sci. Rep. 4, 5663 (2014). 25012721 10.1038/srep05663 PMC4092328

[b7] Micle O., Muresan M., Antal L., Bodog F. & Bodog A. The influence of homocysteine and oxidative stress on pregnancy outcome. J. Med. Life. 5, 68–73 (2012). 22574089 PMC3307082

[b8] Arsenault B. J., Boekholdt S. M. & Kastelein J. J. Lipid parameters for measuring risk of cardiovascular disease. Nat. Rev. Cardiol. 8, 197–206 (2011). 21283149 10.1038/nrcardio.2010.223

[b9] Maxfield F. R. & Tabas I. Role of cholesterol and lipid organization in disease. Nature. 438, 612–621 (2005). 16319881 10.1038/nature04399

[b10] Expert Panel on Detection, Evaluation, and Treatment of High Blood Cholesterol in Adults. Executive summary of the third report of the National Cholesterol Education Program (NCEP) Expert Panel on Detection, Evaluation, and Treatment of High Blood Cholesterol in Adults (Adult Treatment Panel III). JAMA. 285, 2486–2497 (2001). 11368702 10.1001/jama.285.19.2486

[b11] Stone N. J. *et al.* 2013 ACC/AHA guideline on the treatment of blood cholesterol to reduce atherosclerotic cardiovascular risk in adults: a report of the American College of Cardiology/American Heart Association Task Force on Practice Guidelines. Circulation. 129 (suppl 2), S1–S45 (2014). 24222016 10.1161/01.cir.0000437738.63853.7a

[b12] Goff D. C. Jr. *et al.* 2013 ACC/AHA guideline on the assessment of cardiovascular risk: a report of the American College of Cardiology/American Heart Association Task Force on Practice Guidelines. Circulation. 129 (Suppl 2), S49–S73 (2014). 24222018 10.1161/01.cir.0000437741.48606.98

[b13] Kannel W. B. Lipids, diabetes, and coronary heart disease: insights from the Framingham Study. Am. Heart J. 110, 1100–1107 (1985). 4061265 10.1016/0002-8703(85)90224-8

[b14] Ingelsson E. *et al.* Clinical utility of different lipid measures for prediction of coronary heart disease in men and women. JAMA. 298, 776–785 (2007). 17699011 10.1001/jama.298.7.776

[b15] Lee Y. H. & Song G. G. Pathway analysis of genome-wide association studies on uric acid concentrations. Hum. Immunol. 73, 805–810 (2012). 22609445 10.1016/j.humimm.2012.05.004

[b16] Reimer R. J. SLC17: a functionally diverse family of organic anion transporters. Mol. Aspects Med. 34, 350–359 (2013). 23506876 10.1016/j.mam.2012.05.004 PMC3927456

[b17] Busch A. E. *et al.* Expression of a renal type I sodium/phosphate transporter (NaPi-1) induces a conductance in Xenopus oocytes permeable for organic and inorganic anions. Proc. Natl. Acad. Sci. USA. 93, 5347–5351 (1996). 8643577 10.1073/pnas.93.11.5347 PMC39248

[b18] Urano W. *et al.* Sodium-dependent phosphate cotransporter type 1 sequence polymorphisms in male patients with gout. Ann. Rheum. Dis. 69, 1232–1234 (2010). 19556210 10.1136/ard.2008.106856

[b19] Roddy E., Mallen C. D., Hider S. L. & Jordan K. P. Prescription and comorbidity screening following consultation for acute gout in primary care. Rheumatology (Oxford). 49, 105–111 (2010). 19920095 10.1093/rheumatology/kep332

[b20] Kolz M. *et al.* Meta-analysis of 28,141 individuals identifies common variants within five new loci that influence uric acid concentrations. PLoS Genet. 5, e1000504 (2009). 19503597 10.1371/journal.pgen.1000504 PMC2683940

[b21] Yang Q. *et al.* Multiple genetic loci influence serum urate levels and their relationship with gout and cardiovascular disease risk factors. Circ. Cardiovasc. Genet. 3, 523–530 (2010). 20884846 10.1161/CIRCGENETICS.109.934455 PMC3371395

[b22] Ioachimescu A. G., Brennan D. M., Hoar B. M., Hazen S. L. & Hoogwerf B. J. Serum uric acid is an independent predictor of all-cause mortality in patients at high risk of cardiovascular disease: a preventive cardiology information system (PreCIS) database cohort study. Arthritis Rheum. 58, 623–630 (2008). 18240236 10.1002/art.23121

[b23] Ridker P. M. LDL cholesterol: controversies and future therapeutic directions. Lancet. 384, 607–617 (2014). 25131980 10.1016/S0140-6736(14)61009-6

[b24] LaRosa J. C. *et al.* Intensive lipid lowering with atorvastatin in patients with stable coronary disease. N. Engl. J. Med. 352, 1425–1435 (2005). 15755765 10.1056/NEJMoa050461

[b25] Nicholls S. J. *et al.* Statins, high-density lipoprotein cholesterol, and regression of coronary atherosclerosis. JAMA. 297, 499–508 (2007). 17284700 10.1001/jama.297.5.499

[b26] Choi S. T., Kim J. S. & Song J. S. Elevated serum homocysteine levels were not correlated with serum uric acid levels, but with decreased renal function in gouty patients. J. Korean Med. Sci. 29, 788–792 (2014). 24932079 10.3346/jkms.2014.29.6.788 PMC4055811

[b27] Liao D. *et al.* Hyperhomocysteinemia decreases circulating high-density lipoprotein by inhibiting apolipoprotein A-I Protein synthesis and enhancing HDL cholesterol clearance. Circ. Res. 99, 598–606 (2006). 16931800 10.1161/01.RES.0000242559.42077.22 PMC4400841

[b28] Obeid R. & Herrmann W. Homocysteine and lipids: S-adenosyl methionine as a key intermediate. FEBS. Lett. 583, 1215–1225 (2009). 19324042 10.1016/j.febslet.2009.03.038

[b29] Kario K. *et al.* High plasma homocyst(e)ine levels in elderly Japanese patients are associated with increased cardiovascular disease risk independently from markers of coagulation activation and endothelial cell damage. Atherosclerosis. 157, 441–449 (2001). 11472745 10.1016/s0021-9150(00)00738-3

[b30] Olthof M. R., van Vliet T., Verhoef P., Zock P. L. & Katan M. B. Effect of homocysteine-lowering nutrients on blood lipids: results from four randomised, placebo-controlled studies in healthy humans. PLoS Med. 2, e135 (2005). 15916468 10.1371/journal.pmed.0020135 PMC1140947

[b31] Lonn E. *et al.* Homocysteine lowering with folic acid and B vitamins in vascular disease. N. Engl. J. Med. 354, 1567–1577 (2006). 16531613 10.1056/NEJMoa060900

[b32] Taniguchi A. & Kamatani N. Control of renal uric acid excretion and gout. Curr. Opin. Rheumatol. 20, 192–197 (2008). 18349750 10.1097/BOR.0b013e3282f33f87

[b33] Culleton B. F., Larson M. G., Kannel W. B. & Levy D. Serum uric acid and risk for cardiovascular disease and death: the Framingham Heart Study. Ann. Intern. Med. 131, 7–13 (1999). 10391820 10.7326/0003-4819-131-1-199907060-00003

[b34] Hamajima N. The Japan Multi-Institutional Collaborative Cohort Study (J-MICC Study) to detect gene-environment interactions for cancer. Asian Pac. J. Cancer Prev. 8, 317–323 (2007). 17696755

[b35] Wakai K. *et al.* Profile of participants and genotype distributions of 108 polymorphisms in a cross-sectional study of associations of genotypes with lifestyle and clinical factors: a project in the Japan Multi-Institutional Collaborative Cohort (J-MICC) Study. J. Epidemiol. 21, 223–235 (2011). 21467728 10.2188/jea.JE20100139 PMC3899413

[b36] Friedewald W. T., Levy R. I. & Fredrickson D. S. Estimation of the concentration of low-density lipoprotein cholesterol in plasma, without use of the preparative ultracentrifuge. Clin. Chem. 18, 499–502 (1972). 4337382

[b37] Imaeda N. *et al.* Reproducibility of a short food frequency questionnaire for Japanese general population. J. Epidemiol. 17, 100–107 (2007). 17545697 10.2188/jea.17.100 PMC7058456

[b38] Tokudome Y. *et al.* Relative validity of a short food frequency questionnaire for assessing nutrient intake versus three-day weighed diet records in middle-aged Japanese. J. Epidemiol. 15, 135–145 (2005). 16141632 10.2188/jea.15.135 PMC7851066

[b39] Tokudome S. *et al.* Development of a data-based short food frequency questionnaire for assessing nutrient intake by middle-aged Japanese. Asian Pac. J. Cancer Prev. 5, 40–43 (2004). 15075003

[b40] Goto C. *et al.* Validation study of fatty acid consumption assessed with a short food frequency questionnaire against plasma concentration in middle-aged Japanese people. Scand. J. Nutr. 50, 77–82 (2006).

[b41] Hara M. *et al.* Effect of the PPARG2 Pro12Ala polymorphism and clinical risk factors for diabetes mellitus on HbA1c in the Japanese general population. J. Epidemiol. 22, 523–531 (2012). 23006958 10.2188/jea.JE20120078 PMC3798564

[b42] Ohnishi Y. *et al.* A high-throughput SNP typing system for genome-wide association studies. J. Hum. Genet. 46, 471–477 (2001). 11501945 10.1007/s100380170047

